# Electronic states of deep trap levels in a-plane GaN templates grown on r-plane sapphire by HVPE

**DOI:** 10.1038/s41598-018-26290-y

**Published:** 2018-05-18

**Authors:** Moonsang Lee, Thi Kim Oanh Vu, Kyoung Su Lee, Eun Kyu Kim, Sungsoo Park

**Affiliations:** 1 Korea Basic Science Institute, 169-148 Gwahak-ro, Yuseong-gu, Daejeon, Republic of Korea; 2 Quantum-Function Research Laboratory, Hanyang University, Department of Physics, Seoul, 133-791 Republic of Korea; 3 Department of Science Education, Jeonju University, 303 Cheonjam-ro, Wansan-gu, Jeollabuk-do Republic of Korea; 4 Analytical Laboratory of Advanced Ferroelectric Crystals, Jeonju University, 303 Cheonjam-ro, Wansan-gu, Jeollabuk-do Republic of Korea

## Abstract

We report on the defect states incorporated in a-plane GaN crystals grown on r-plane sapphire substrates by hydride vapor phase epitaxy (HVPE), using deep level transient spectroscopy (DLTS). Two defect states were observed at 0.2 eV and 0.55 eV below the conduction band minimum with defect densities of 5 × 10^12^/cm^3^ and 4.7 × 10^13^/cm^3^, respectively. The size of capture cross section, non-linear relation of trap densities from the depth profile, filling pulse width, and PL measurements indicated that the electronic deep trap levels in a-plane GaN on r-plane sapphire by HVPE originated from non-interacting point defects such as N_Ga_, complex defects involving Si, O, or C, and V_Ga_-related centres. Even though the a-plane GaN templates were grown by HVPE with high growth rates, the electronic deep trap characteristics are comparable to those of a-plane GaN layers of high crystal quality grown by MOCVD. This study prove that the growth of a-plane GaN templates on r-plane sapphire by HVPE is a promising method to obtain a-plane GaN layers efficiently and economically without the degradation of electrical characteristics.

## Introduction

Group III-nitrides and their compounds have been significantly considered as adequate materials for opto-electronic applications. Although a great device performance has been achieved for gallium nitride (GaN)-based light-emitting diodes (LEDs), the conventional GaN-based devices with c-polarity suffer from a quantum-confined stark effect (QCSE) along their orientations that is induced by spontaneous polarisation and a piezoelectric field. This leads to a large spatial separation between the electron and hole wave functions, resulting in the dissipation of the internal quantum efficiency in quantum well layers^1–3^. Meanwhile, it is well known that this negative effect of polar GaN-based devices can be made negligible by the use of non-polar GaN layers^4,5^. Owing to this expectation, the research on non-polar GaN has gained considerable attention recently^6,7^.

Researchers have studied the growth of non-polar GaN using various techniques. One of the well-known methods for fabricating non-polar GaN substrates is to slice bulk polar GaN along the non-polar direction^8^. However, this method is not appropriate for commercial purposes because of its low productivity and size limitation. The most feasible, popular, and commercially available method for non-polar GaN substrate fabrication is heteroepitaxially growing non-polar GaN on foreign substrates, using metal organic chemical vapour deposition (MOCVD), molecular beam epitaxy (MBE), or hydride vapor-phase epitaxy (HVPE)^9–11^. Among these methods, HVPE is the most promising because it provides a high throughput and relatively good crystal quality. However, the opto-electrical and structural properties of non-polar GaN layers grown by HVPE with high growth rates could be worse than those of the GaN-layers grown by MOCVD. Nevertheless, the growth of non-polar thick GaN layers on sapphire by HVPE has been considered owing to its high throughput^12^. In spite of the importance given to the non-polar GaN layers grown by HVPE, little has been known about their electronic deep level states. To better understand their states, it is required to understand, identify, and characterise the defects embedded in the non-polar GaN layers grown by HVPE; this can help optimise the performance of non-polar GaN-based devices.

In this study, we employed deep level transition spectroscopy measurements to investigate the electrically activated defect states of a-plane GaN layers grown on r-plane sapphire by HVPE.

## Results and Discussion

**Figure 1:**
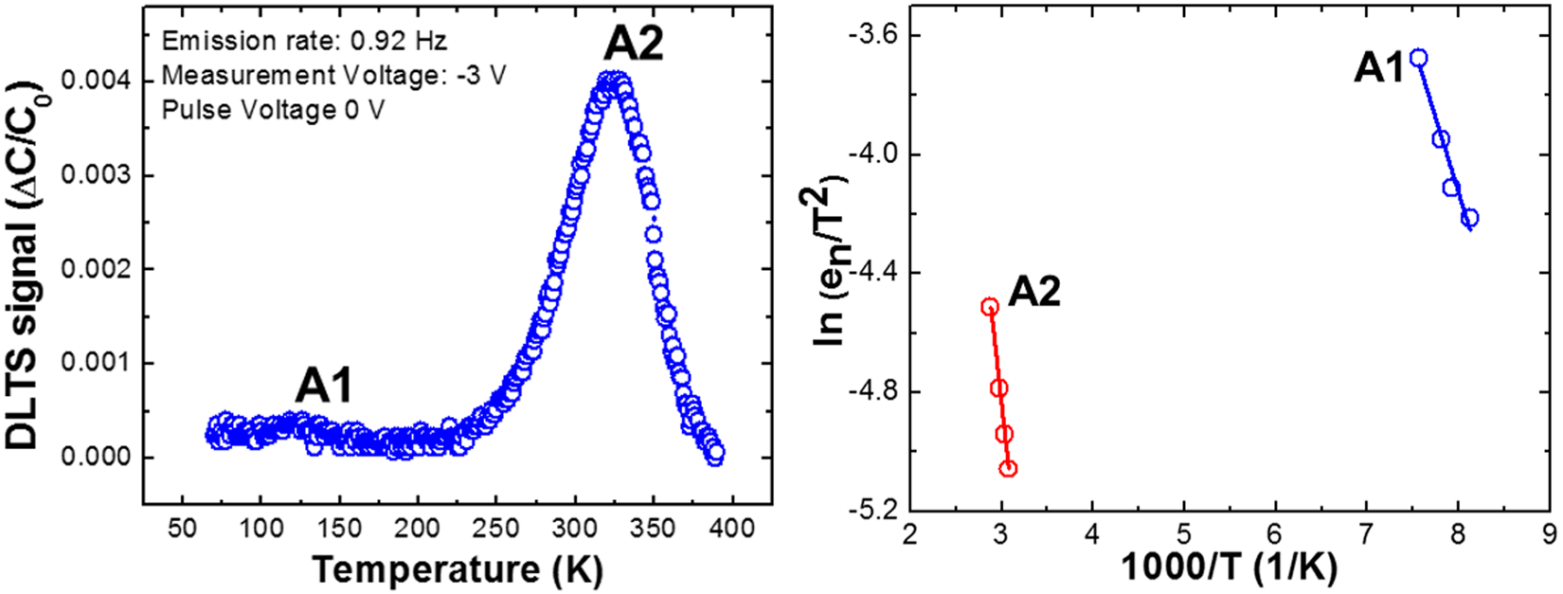
(**a**) DLTS spectrum of a-plane GaN layers on r-plane sapphire substrates measured at pulse voltage of 0 V and reverse voltage of −3 V in the temperature range from 70 to 370 K. (**b**) Arrhenius plots of DLTS signals for a-plane GaN layers on r-plane sapphire substrates.

Figure 1(a,b) show the DLTS spectra and Arrhenius plots respectively of the defect states in a-plane GaN layers grown on r-plane sapphire substrates by HVPE. Here, the DLTS signal from 70 K to 370 K was measured at an emission rate (e_n_) of 0.92 Hz under an applied filling pulse width of 15 ms, where the pulse voltage is 0 V and the reverse bias voltage is −3 V. Two DLTS signals are clearly shown, and labelled as A1 and A2.

From the Arrhenius plot shown in Fig. 1(a), the defect parameters such as activation energy (E_a_), capture cross section (σ_n_), and defect density of A1 and A2 were obtained and have been summarised in Table 1. The E_a_ and σ_n_ of the defect states can be calculated from the emission rate equation^13^:

1 $$\mathrm{ln}(\frac{{{\rm{e}}}_{{\rm{n}}}}{{{\rm{T}}}^{2}})=\,\mathrm{ln}(\sqrt{6}{\rm{\pi }}1.5{k}^{2}{{\rm{m}}}_{{\rm{n}}}^{\ast }{{\rm{\sigma }}}_{{\rm{n}}}/{{\rm{h}}}^{3})+(-{\rm{\Delta }}{{\rm{E}}}_{{\rm{a}}}/1000\,{\rm{k}})1000/{\rm{T}}$$

where e_n_ is the emission rate, T the absolute temperature, k Boltzmann’s constant, m^*^ effective mass of the carrier, and h Plank’s constant.

**Table 1:** Defect parameters for a-plane GaN layers on r-plane sapphire substrates by HVPE.

Defect	Activation energy (eV)	Capture cross section (cm^2^)	Trap density (cm^−3^)
A1	0.2	1.14 × 10^−17^	5 × 10^12^
A2	0.55	4.4 × 10^−17^	4.7 × 10^13^

In addition, the trap densities can be evaluated as follows^14^:

2 $${{\rm{N}}}_{{\rm{t}}}=2{{\rm{N}}}_{{\rm{d}}}{{\rm{F}}}^{-1}{\rm{\Delta }}C/{\rm{C}}$$

where N_d_ is the donor concentration, ∆C is the capacitance change during relaxation and F is the spectrometer function of 3.5 used in this work. The two defect levels of A1 and A2 are positioned at 0.2 eV and 0.55 eV below the conduction band, respectively. The defect A2 (E_c_ − 0.55 eV) is the dominant defect with a defect density of 4.7 × 10^13^/cm^3^ and a capture cross section of 4.4 × 10^−17^ cm^2^. Meanwhile, the trap A1 with a relatively low DLTS signal has a density of 5.0 × 10^12^/cm^3^ and capture cross section 1.14 × 10^−17^ cm^2^. These low trap densities are comparable to those of a-plane GaN layers with high crystal quality grown by MOCVD^13,15,16^. Considering this, we speculate that a-plane GaN templates grown by HVPE possess a desirable advantage in view of throughput for the formation of opto-electrical devices, compared to those grown by MOCVD.

The detected trap level A1 (Ec − 0.2 eV) and the level A2 (Ec − 0.55 eV) are commonly observed in the other literatures regardless of the growth method and orientation^17–19^. The A1 trap has been reported to relate to the native defects that strongly bonded to threading dislocations^20^, which are composed of complex defects with Ga vacancy (V_Ga_) such as V_N_-V_Ga_^21^. Furthermore, the trap A2 can be considered as point defects associated with nitrogen anti-site (N_Ga_)^22^, complex defects involving Si, O, or C^23^ or intrinsic defects^24^.

**Figure 2:**
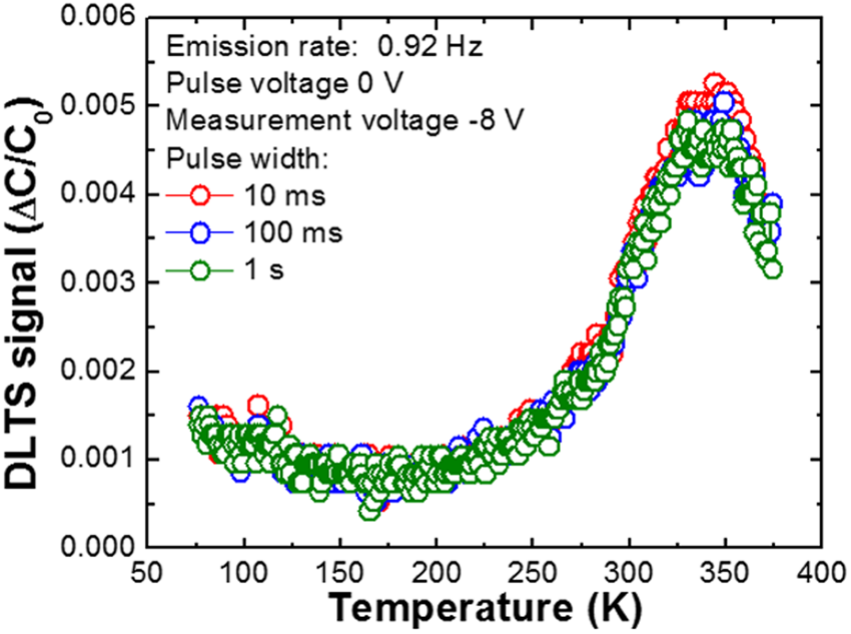
DLTS spectra of a-plane GaN layer on r-plane sapphire measured at e_n_ of 0.92 Hz under different filling pulse width, where pulse voltage is 0 V and measurement voltage −8 V.

To clarify the origin of the deep trap levels in the a-plane GaN layers grown on r-plane sapphire by HVPE, we investigated the DLTS signals as a function of the filling pulse width (t_p_), as has been described in Fig. 2. It is essential to note that the DLTS signal is independent of the filling pulse width. Indeed, defects can be classified into non-interacting and interacting types. The non-interacting defects such as point defects do not influence each other. On the other hand, the interacting defects such as dislocations and stacking faults affect carrier capture and emission in the surrounding regions. It is well known that trap occupancy in the case of non-interacting defects depends exponentially on t_p_ while that of interacting defects are proportional to ln(t_p_)^13^. Because a plot of a DLTS signal as a function of ln(t_p_) should produce a straight line for linearly arranged interacting defects and a non-linear curve for non-interacting defects, a correlation between the extended defects and deep levels can be identified. From the results of Fig. 2, it is found that A2 can be classified as a point defect because the DLTS signals of A1 and A2 are independent of t_p_.(See Fig. S1) In addition, considering the size of cross-section of the defects A1 and A2, it can be concluded that both A1 and A2 are point and not dislocation defects.

**Figure 3:**
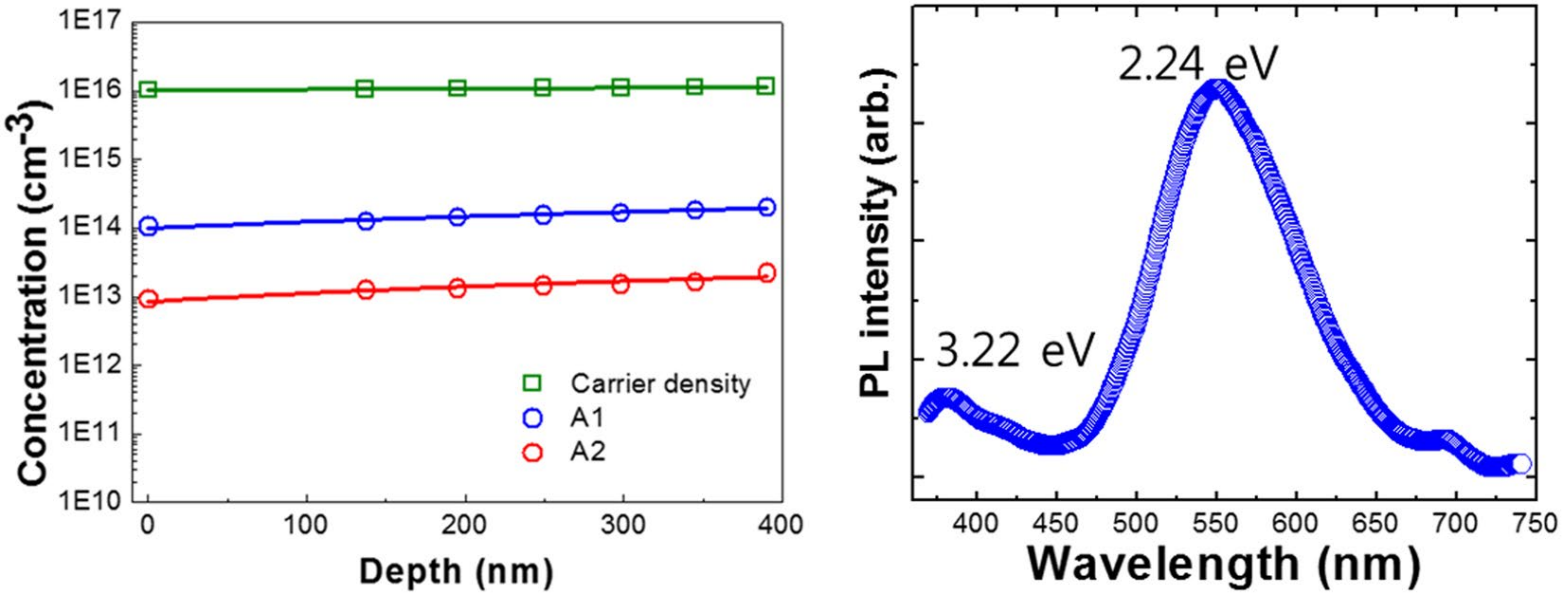
(**a**) Depth profiles of carrier concentration, and trap A1 and A2 under various applied voltage, and (**b**) room temperature PL spectrum of a-plane GaN layer on r-plane sapphire.

Figure 3(a) shows the defect density and carrier concentration of A1 and A2 in the a-plane GaN layer as a function of depth under different applied reverse bias conditions from −3 V to −9 V that are obtained from the DLTS signal and capacitance-voltage profile, respectively. The reverse bias condition determines the measurement depth from the surface of an a-plane GaN layer. In other words, when a higher reverse bias is applied to the a-plane GaN layer, the defect and the carrier concentration appear at a deeper depth. As shown in Fig. 3(a), the signal corresponding to the defect was almost constant through the sample depth. Considering the non-linear relation between the DLTS signal and ln (t_p_) and capture cross-section (~10^17^ cm^2^) of A1 and A2, it is concluded that the origin of these defects is related to the non-interacting point defects. Furthermore, from the photoluminescence (PL) measurement at room temperature shown in Fig. 3(b), the a-plane GaN shows a broad band emission near 2.24 eV, which is attributed to yellow defect luminescence with a FWHM of about 100 nm. Lyons *et al*. computed that V_Ga_ and substitutional oxygen (O_N_) complex may give rise to yellow luminescence near an emission peak of ~2.2 eV, using density-functional theory (DFT)^25^. Furthermore, Neugebauer *et al*. reported that yellow luminescence results from the V_Ga_-O_N_ complex that is formed during high temperature growth^26^. It is well known that O_N_ is common in n-GaN as a dopant or impurity^27^. Note that the typical GaN layers grown by HVPE include oxygen concentration owing to an unintentional doping by quartz tube and residual oxygen in the HVPE reactor. Therefore, the result of Fig. 3(b) is consistent with the DLTS results that A2 is dominant defect associated with complex defects involving oxygen, and the previous reports.

## Conclusions

In summary, DLTS analysis was employed to investigate the electronic trap levels in a-plane GaN grown on r-plane sapphire by HVPE. Two electron traps at Ec − 0.2 eV and −0.55 eV with densities of 5 × 10^12^/cm^3^ and 4.7 × 10^13^/cm^3^ were documented for the a-plane GaN grown on r-plane sapphire by HVPE; the traps have capture cross sections of 1.14 × 10^−17^ cm^2^ and 4.4 × 10^−17^ cm^2^ respectively. The size of capture cross sections, non-linear variation of defect densities (from the depth profile), filling pulse width, and PL measurements confirmed that the defect states originated from non-interacting point defects such as N_Ga_, complex defects involving Si, O or C and V_Ga_-related centres. Even though the a-plane GaN layers were grown by HVPE with high growth rates, which can result in low crystal qualities, the characteristics of their defect states are compared to those of a-plane GaN with high crystal quality grown using MOCVD. We believe that the growth of a-plane GaN templates on r-plane sapphire by HVPE is a promising method for the development of non-polar GaN-based high-performance devices.

## Methods

Non-polar a-plane GaN layers were grown on 2-inch (1–102) r-plane sapphire substrates using a vertical-type downstream HVPE system with a reactor diameter of 6 inches. To prevent the development of significant stresses during non-polar GaN growth on the substrates, the substrates were subjected to a surface treatment before growth. The treatment consisted of HCl etching (flow rate: 70 sccm) and nitridation with NH_3_ gas (flow rate: 2500 sccm) for 5 min. This treatment resulted in the formation of a discontinuous GaN nanodot buffer layer on the substrates^28^. Scanning electron microscopy (SEM) analysis revealed the discontinuous layer to be composed of GaN nanodots (not shown in this paper). Subsequently, GaN was grown on the GaN nandot buffer layer under atmospheric pressure and temperature ~1080 °C. The HCl gas (flow rate: 40 sccm) was reacted with liquid Ga metal to form GaCl gas (conversion efficiency ~15%). Subsequently, GaCl was transported to the growth zone where it reacted with NH_3_, leading to GaN layer growth. The total flow rate of N_2_ used as the carrier gas was 15000 sccm and the V/III ratio was approximately 6. The thickness of the grown non-polar a-plane GaN layers was 5 µm. The densities of stacking fault and threading dislocation were 4.12 × 10^5^/cm, and 3.86 × 10^9^/cm^2^, respectively. (Not shown in this paper) These values are comparable to those grown by MOCVD^29,30^.

For the electrical characterisation of a-plane GaN grown on r-plane sapphire using HVPE, Al(150 nm) layers of 3 mm diameter were deposited on a-plane GaN as ohmic contacts using a thermal evaporator. The ohmic contacts were then annealed at 550 °C in an Ar ambient to improve contact formation. Subsequently, Schottky contacts of 300 µm diameter were fabricated by an electron beam evaporator using Au (40 nm) on the a-plane GaN layers, followed by rapid thermal annealing in Ar at 550 °C. The measurements of the deep level traps in the a-plane GaN layers grown on r-plane sapphire by HVPE were performed in an indigenously developed system, using a 100 mV signal at 1 MHz in the temperature range 70–370 K. In addition, the optical properties of the a-plane GaN layers grown on r-plane sapphire using HVPE were determined by PL measurements using a He–Cd laser of excitation wavelength 325 nm and output power 10 mW at room temperature.

## Electronic supplementary material

Supplementary Information

## References

[1] Chung K (2014). Growth and characterizations of GaN micro-rods on graphene films for flexible light emitting diodes. Apl Materials.

[2] Shih H-Y (2015). Ultralow threading dislocation density in GaN epilayer on near-strain-free GaN compliant buffer layer and its applications in hetero-epitaxial LEDs. Scientific reports.

[3] Suihkonen S, Pimputkar S, Speck JS, Nakamura S (2016). Infrared absorption of hydrogen-related defects in ammonothermal GaN. Applied Physics Letters.

[4] Ma H (2011). GaN crystals prepared through solid-state metathesis reaction from NaGaO2 and BN under high pressure and high temperature. Journal of Alloys and Compounds.

[5] Takeuchi S (2017). Control of dislocation morphology and lattice distortion in Na-flux GaN crystals. Journal of Applied Physics.

[6] Cich MJ (2012). Bulk GaN based violet light-emitting diodes with high efficiency at very high current density. Applied Physics Letters.

[7] Jachalke S (2016). The pyroelectric coefficient of free standing GaN grown by HVPE. Applied Physics Letters.

[8] Paskova T (2007). Nonpolar a-and m-plane bulk GaN sliced from boules: structural and optical characteristics. physica status solidi (c).

[9] Able A, Wegscheider W, Engl K, Zweck J (2005). Growth of crack-free GaN on Si(1 1 1) with graded AlGaN buffer layers. J. Cryst. Growth..

[10] Armitage R (2002). Lattice-matched HfN buffer layers for epitaxy of GaN on Si. Appl. Phys. Lett..

[11] Arslan E, Ozturk MK, Teke A, Ozcelik S, Ozbay E (2008). Buffer optimization for crack-free GaN epitaxial layers grown on Si (1 1 1) substrate by MOCVD. Journal of Physics D: Applied Physics.

[12] Yang J (2015). Green light emitting diode grown on thick strain-reduced GaN template. Materials Science in Semiconductor Processing.

[13] Pak SW (2013). Defect states of a-plane GaN grown on r-plane sapphire by controlled integration of silica nano-spheres. Journal of Crystal Growth.

[14] Arehart AR, Allerman AA, Ringel SA (2011). Electrical characterization of n-type Al0.30Ga0.70N Schottky diodes. J. Appl. Phys..

[15] Song H (2012). Characterization of deep levels in a-plane GaN epi-layers grown using various growth techniques. J. Cryst. Growth..

[16] Iida D (2010). Compensation effect of Mg-doped a- and c-plane GaN films grown by metalorganic vapor phase epitaxy. J. Cryst. Growth..

[17] Nguyen XS (2016). Deep level traps in semi-polar n-GaN grown on patterned sapphire substrate by metalorganic vapor phase epitaxy. Phys. Stat. Solidi b.

[18] Duc TT, Pozina G, Janzen E, Hemmingsson C (2013). Investigation of deep levels in bulk GaN material grown by halide vapor phase epitaxy. J. Appl. Phys..

[19] PŁaczek-Popko E (2009). Deep level transient spectroscopy signatures of majority traps in GaN p–n diodes grown by metal-organic vapor-phase epitaxy technique on GaN substrates. Physica B: Condensed Matter.

[20] Soh CB (2004). Assignment of deep levels causing yellow luminescence in GaN. J. Appl. Phys..

[21] Fang Z-Q (2001). Evolution of deep centers in GaN grown by hydride vapor phase epitaxy. Appl. Phys. Lett..

[22] Cho HK, Kim CS, Hong C-H (2003). Electron capture behaviors of deep level traps in unintentionally doped and intentionally doped n-type GaN. J. Appl. Phys..

[23] Look DC, Fang Z-Q, Claflin B (2005). Identification of donors,acceptors,and traps in bulk-like HVPE GaN. J. Cryst. Growth..

[24] Armstrong A (2005). Impact of deep levels on the electrical conductivity and luminescence of gallium nitride codoped with carbon and silicon. J. Appl. Phys..

[25] Lyons JL, Alkauskas A, Janotti A, Van de Walle CG (2015). First-principles theory of acceptors in nitride semiconductors. physica status solidi (b).

[26] Neugebauer J, de Walle CGV (1996). Gallium vacancies and the yellow luminescence in GaN. Appl. Phys. Lett..

[27] Xie, Z., *et al* Assignment of multiband luminescence due to the gallium vacancy-oxygen defect complex inGaN. *arXiv preprint arXiv:1802.1022*2 (2018).

[28] Lee M, M. D, Park S (2017). Thick GaN growth via GaN nanodot formation by HVPE. CrystEngComm..

[29] Moram M, Johnston C, Kappers M, Humphreys C (2010). Measuring dislocation densities in nonpolar a-plane GaN films using atomic force microscopy. Journal of Physics D: Applied Physics.

[30] Tuomisto F (2007). Defect distribution in a-plane GaN on Al 2 O 3. Applied physics letters.

