# Supplementary material for: Electronic states of deep trap levels in a-plane GaN templates grown on r-plane sapphire by HVPE
Source: Sci Rep. 2018 May 18;8:7814. doi: 10.1038/s41598-018-26290-y (PMC5959929; doi:10.1038/s41598-018-26290-y)
Supplement: Supplementary file 1 — Supplementary Information [file 41598_2018_26290_MOESM1_ESM.docx]

**Supplementary information**

**Electronic states of deep trap levels in a-plane GaN templates grown on r-plane sapphire by HVPE**

# Moonsang Lee1,*, Thi Kim Oanh Vu2, Kyoung Su Lee^2^, Eun Kyu Kim2,*, and Sungsoo Park3,4,*

^1^Korea Basic Science Institute, 169-148, Gwahak-ro, Yuseong-gu, Daejeon, Republic of Korea

^2^Quantum-Function Research Laboratory, Hanyang University, Department of Physics, Seoul 133-791, Republic of Korea

^3^Department of Science Education, Jeonju University, 303 Cheonjam-ro, Wansan-gu, Jeollabuk-do, Republic of Korea

^4^Analytical Laboratory of Advanced Ferroelectric Crystals, Jeonju University, 303 Cheonjam-ro, Wansan-gu, Jeollabuk-do, Republic of Korea

*[lms1015@kbsi.re.kr, ek-kim@hanyang.ac.kr,](mailto:corresponding.author@email.example) sspark@jj.ac.kr


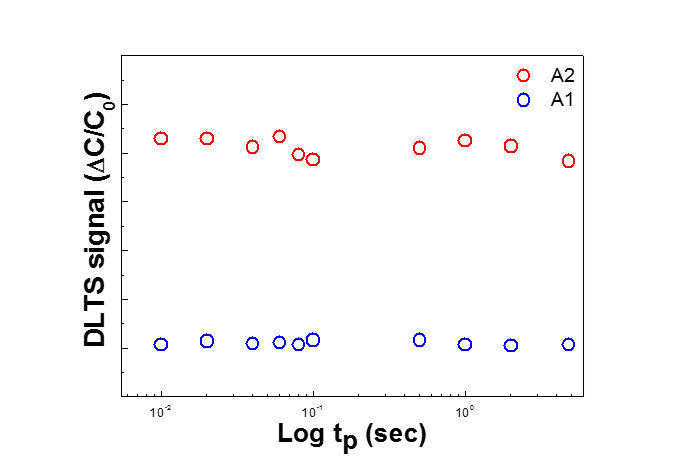


**Fig. S1** DLTS signals of traps A1, and A2 vs. filling pulse width, t_p_.
